# Human Adaptation to Deep Space Environment: An Evolutionary Perspective of the Foreseen Interplanetary Exploration

**DOI:** 10.3389/fpubh.2020.00119

**Published:** 2020-04-24

**Authors:** François Criscuolo, Cédric Sueur, Audrey Bergouignan

**Affiliations:** ^1^Université de Strasbourg, CNRS, IPHC UMR 7178, Strasbourg, France; ^2^Division of Endocrinology, Metabolism and Diabetes, Anschutz Health & Wellness Center, University of Colorado, Anschutz Medical Campus, Aurora, CO, United States

**Keywords:** physiology, human, adaptation, evolution, space

## Abstract

Long-term and deep space exploration is a prevailing dream that is becoming a reality. Is that so? The answer to this question depends on how the main actors of space exploration, i.e., politicians, scientists, and engineers, define “long-term” and the ultimate goals of the current space programs. Presently, long-term refers to few months or years, which is equivalent to the time necessary for a manned mission to reach another planet and return to Earth. Such a space mission is a tremendous scientific challenge associated with multidisciplinary issues spanning from technology to medicine biology, social, and psychological science. It has been a priority of the main westernized societies that has attracted the brightest and most innovative scientific minds since World War II. At first the stakes were mainly political in order to demonstrate to other countries power and strength. It progressively became a scientific motivation to uncover the secrets of the Universe and life's origin, and potentially to find traces of distant life. More recently, a desire to colonize space and exploit resources on other planets has emerged as a new dream. Although the journey to Mars is still a prospective and traveling in deep space a further elusive goal, one can question the ultimate implications of deep space exploration over the long-term.

Long-term and deep space exploration is a prevailing dream that is becoming a reality. Is that so? The answer to this question depends on how the main actors of space exploration, i.e., politicians, scientists, and engineers, define “long-term” and the ultimate goals of current space programs. Presently, long-term refers to few months or years, which is equivalent to the time necessary for a manned mission to reach another planet and return to Earth. Such a space mission is a tremendous scientific challenge associated with multidisciplinary issues spanning from technology (1) to medicine biology (2), social and psychological science (3). It has been a priority of main westernized societies that have attracted the brightest and most innovative scientific minds since World War II. At first, the stakes were mainly political in order to demonstrate to other countries both power and strength. It progressively became a scientific motivation to uncover the secrets of the Universe and life's origin, and potentially to find traces of distant life. More recently, a desire to colonize space and exploit resources on other planets emerged as a new dream. Although the journey to Mars is still a prospective goal and traveling into deep space is an elusive goal (4), one can question the implications of deep space exploration over the long-term.

This perspective requires subscribing to a new paradigm that no longer sees “long-term” as months or years but rather as time in an evolutionary context. This means that instead of thinking about the physiological and psychological response of the human body to the space environment, we must consider the adaptations that will be naturally selected by this extreme environment. The long-term objective may then be to provide humanity an access to space shelters (i.e., spaceships or exoplanets) in order to survive the Sun's death.

Traveling into deep space should also be a concern for evolutionary biology and ecology research fields. Including evolutionary concepts to better assess the long-term challenges imposed by the presence of humans in space could open up new perspectives for imagining how future successful generations of humans will cope with the environmental conditions of space. This type of question belongs to the research field of evolutionary biology, which essentially tackles how evolution resolves previous challenges imposed to life on Earth. We believe this question easily extends to how evolution will help a human population adapt to an environment that is drastically different from the present on Earth. In fact, evolution through natural selection has led to the emergence of species that can live in extreme environments. Some prokaryotic microorganism (e.g., bacteria), crabs and fishes can inhabit extreme environments like boiling waters and/or live under high environmental pressure. Some vertebrates (mammals and birds) can also live when facing ambient temperatures of −40°C or sustaining highly-demanding physical activities at an altitude above 7,000 m. Although not presented in the present perspective, these types of questions on the evolutionary mechanisms and environmental limits of living beings were recognized by the NASA Astrobiology Roadmap as one of the scientific objectives to be addressed (5).

Research in space life science predominantly focuses on understanding the physiological adaptations to the space environment, i.e., physiological responses to microgravity and radiation, and to a lesser extent, the loss of nycthemeral cycles, exposure to extreme temperatures or hypercapnic conditions present in the International Space Station (ISS). The goal is to assess the impact of these changes on health and consequently, on the safety and survival of the crew members. It is well-known that microgravity leads to a myriad of body alterations including bone and muscle mass loss, cardiovascular deconditioning, impaired exercise capacity, immune-deficiency, and alterations of peripheral metabolism (6–8). To prevent the development of these physiological modifications during spaceflights, international space agencies have put a lot of effort into the development of countermeasures. Countermeasure programs essentially consistof nutritional and pharmacological treatments, exercise training protocols, vibrations and low body negative pressure, either used separately or in combination with each other (2). Adaptations to the space environment are often referred to as maladaptations when they are, in fact, physiological responses to a new environment with different physical characteristics. What is commonly considered maladaptive is a physiological trait that deviates from an optimal response shaped by natural selection in the terrestrial environmental conditions, but not an inability to adapt to space environment. A first provisional response to such a challenge could be to artificially modify the human physiology to allow human life to thrive in the unique space environment. One could imagine that synthetic molecules could be developed to prevent short-term physiological alterations. If long-term administration of synthetic molecules does not trigger additional medical issues, this could be a promising avenue for space research on human adaptation (9). Different approaches developed by the field of synthetic biology (10), such as genetic engineering or synthetic molecules redefining the main physiological pathways could theoretically provide biological tools for a short-term adaptation to multiple challenges imposed by spaceflight. However, apart from the obvious ethical issues of human design, the start of a new human lineage is not, in our opinion, a definitive solution. Pre-adaptations to space should be based on our current knowledge regarding the health problems associated with astronauts (e.g., bone and muscle loss) which may not be the main limiting factors for the long-term survival of humans in space. Furthermore, exposing these humans designed for living in deep space does not preclude human physiology to pursue an evolutionary process through selection. Nevertheless, synthetic biology offers interesting opportunities. It could be used to either investigate synthetic genetic systems that can neutralize the evolution of key genes, or to send synthetic entities capable of evolution into deep space and thus, ensuring space observation, analysis or pioneering tasks (10).

An alternative is to look at the short-term human physiological response to space in an evolutionary context. We should consider three possibilities when analyzing the unhealthy output of exposition to microgravity. Firstly, not everything in evolution is adaptive. Some of the genetic and phenotypic traits that we observe are the results from the best of misuse strategies. There are many examples in evolution showing that some behaviors, some reproductive tactics, or some phenotypes originated from genetic conflicts or life-history trade-offs, which precludes organisms from perfectly adapting to their environment (11). Thus, it can be considered that humans may never optimally adapt to the space environment. Second, the responses of the human body to the space environment may reflect the short-term mismatch between the rapid and drastic changes in environmental conditions, and the concomitant modifications in human physiology (i.e., phenotypic plasticity). However, plasticity is not adaptation, and the evolution of human traits may require a much longer time-scale (i.e., thousands of years at least) to adapt to space conditions. Again, the synthetic biology may putatively accelerate the adaptation process. However, we know that the extent of bone or body mass loss widely varies among astronauts, some showing dramatic variations in their pre- and post-flight values, while others do not (12). This means that there are genotypes and phenotypes within the human population that may offer some degree of short-term resistance to space environment. In evolutionary biology, this corresponds to the concept of reaction norms (the ability for the same genotype to produce different phenotypes under the influence of the environment). We can envisage that the directional selection conducted so far, based on short-term benefits and comprehensive rules of astronaut's safety, experience and productivity, prevented us from screening the whole distribution of human phenotypes/phenotypic plasticity that best matches with rapid exposition to living conditions in space. The recent rise of private companies (e.g., SpaceX, Blue Origin) that aim to open spaceflight to private passengers, i.e., individuals not selected on the basis of strict physical/cognitive performance, could provide an experimental window to test a wider range of human phenotypes in response to the space environment. Thirdly, we could also consider that the short-term responses observed so far in astronauts belong to an adaptation process in the evolutionary sense, i.e., long-term changes that will promote the selection of genetic and phenotypic variations of individuals associated with higher rate of reproductive success in space. We have already seen that these changes are slow in humans for various reasons including the diploid genome, our developmental constrains, and our pace-of-life. As a conclusion, fast changing variables (i.e., what is currently called human space adaptations) may be indicative or not about long-term adaptability (i.e., evolutionary human adaptation). The answer to this question will be unveiled when the impact of short-term adaptations on human fitness will be tested. With this in mind, we can enter into an evolutionary vision of the study of space biology applied to human biology, which has been surprisingly lacking over the past years (13).

It is far from incongruous to think that space and evolution are linked. Going past the billions of generations that separate us from the very first living being that appeared on Earth 4.5 billion years ago, and go back up one more generation, one can feel the thinness of the presence and absence of life. In a similar vein, the Panspermia theory of Richter and Arrhenius was proposed more than a century ago hypothesizing that some forms of life, resistant to space stressors such as outer space or radiations, might have the ability to spread from planets to planets (14, 15). There is now experimental evidence showing that some life forms such as bacteria or tardigrades may survive exposure to space (16–19). This actually opens up exciting avenues of research for human adaptation to space. Two of them have already been assessed because they have short-term implications. First, microgravity through genomic and phenotypic adaptation may enhance the population growth rate of certain bacteria as well as their virulence or resistance to antibiotics (19–22). This has conducted researchers to study how the host-pathogen relationships can be accordingly modified (23). The second (and still related to the former) concerns changes in the microbiome (i.e., the many microorganisms living in the human host) during exposure to microgravity and radiation. The diversity of microbiomes decreases after a spaceflight, which can weaken some healthy functions such as immunity (24). By consequence, maintaining the microbiome during long-duration spaceflight is a major health challenge for astronauts. These changes may be due to (i) a direct causal effect of microgravity on the bacterial populations of the microbiome, or (ii) an indirect effect of spaceflight environment on the host (i.e., astronauts) physiology, such as stress or change in the quality of the diet (25). These modifications in population composition may reflect intimate changes in the gene expression of bacteria (26), pointing out mechanisms of phenotypic plasticity and norms of reactions to space that need to be better understood. What would be the long-term output of having two entities intimately linked physically and physiologically but evolving at very different rates in response to the space environment? It is likely that natural selection will promote a remodeling of the microbiome toward a composition better associated with the greater reproduction success of its host, integrating the prevailing environmental constrains. This means that we cannot interpret, so far, the observed modification of the microbiome as an alteration of an optimal situation, which has evolved under different conditions on Earth. The temptation to explore the biological engineering of the microbiome (27) to establish the evolutionary stability of bacterial populations is interesting. However, we cannot extrapolate that this will provide the human host with a more suitable phenotype over generations of space travelers. Furthermore, the rate of change of the microbiome in humans is likely to be accelerated by our social nature as a species. As suggested by long-term simulation of living conditions in space (28), changes in the microbiome composition are partially driven by social interactions. Sociality matters for long-term space travels (29); for obvious reasons, it is already taken into account when selecting members for a space mission. As the microbiome influences individual behavior *via* the gut-brain connection (30), it also has evolutionary consequences for the space adaptation of human beings. Despite the fact that highly deleterious parasitic organisms favor host-to-host transmission, limiting horizontal transmission between space mission members may be a key factor considering that humans are slowly developing new host-pathogen relationships. This should be taken into account in studies aimed at resolving infection diseases in deep space. Apart from isolating each person from the other, impinging horizontal transmission is a challenging strategy to implement given the operational capabilities of space shuttles. In conclusion, the rapid and low rates of evolution under space conditions apply to cells and whole-organism (31). The adaptation of cells to gravity may or may not favor the adaptation of individuals (i.e., promote reproductive success in space), and we need more long-term data to fully understand the meaning of the short-term dynamics of single cells in response to the space environment.

When considering human adaptation to the space environment, the selection of individuals with the best reproductive success must be a top priority. However, this has both evolutionary and ethical consequences (32). We would like to highlight here key points relating to reproductive success, methodological or theoretical, both placed in the context of evolutionary theory. First, investigating adaptation in an evolutionary perspective calls for studies at the population level, because it will decipher the nature of the phenotype associated with the highest breeding success during spaceflight. This is the most powerful way to assess how organisms, will succeed surviving the space environment. Previous studies in bacteria subjected to microgravity have revealed interesting evolutionary patterns. The bacterial populations exposed to microgravity display increased growth rates suggesting specific adaptations that lead them to overtake the cultures of their terrestrial siblings (22). Among other possibilities and ranging from the differential expression of genes and proteins, alternative splicing (33), or genome size reduction may explain the higher growth yields of space-exposed bacteria. The ultimate costs in terms of persistence of these mutation and/or phenotypes in the long-term remain to be established. To note, the word reproduction here refers to sexual reproduction (i.e., with male and female gametes) and not asexual reproduction as seen with most bacteria. The evolution of humans in the space environment will never return to asexual reproduction due to developmental constraints inherited from the history of human evolution. This is based on the sequential expression of genes inherited from both the father and the mother during embryonic growth.

How does developmental constraints restrain evolution under microgravity is an interesting topic because phenomena like blastula development, is partly governed by gravity (34). Firstly, we need to use sexually reproducing animal models placing them in microgravity and/or space radiation, and then record the short-term changes in pre- and post-natal growth patterns as well as their genomic and phenotypic changes over time. By allowing the population to evolve and establish these changes, in gene frequencies associated with high reproductive success, we can identify key genes and alleles for space adaptations. Secondly, by adopting an evolutionary perspective of human adaptation to the space environment will bring more clarity beyond medical aspects of human reproduction in microgravity (35). As sexual reproduction encompasses processes such as genetic conflict, mate selection and social constraints, we need to integrate specific traits of human biology and evolution into future experiments. For instance, the kin selection theory (36) has yielded important implications for our understanding of sexual reproduction and the evolution of cooperation. Among these, the mother-father conflict is driving the expression of developmental genes, which are involved in the way the fetus will manipulate the mother's investment in reproduction and the outcome being a gain in fetal mass. Males found a benefice in driving genes promoting mass and the survival of the offspring, while females have to found the best trade-off between the cost of their reproduction, their survival, and chances to reproduce again. The way in which the expression of these genes is altered by the space environment is likely to have tremendous consequences on the evolution of the human population over time. For example, theories are emerging on the relationship between the mother-father conflict and mental illness in offspring (37). Whether autism or schizophrenia prevalence may differ in a space-based human population compared to an Earth-based human population, considering parental conflict or changes in the microbiome (38) has an important predictive value.

Beyond the technological challenges, the question of human presence within in deep space turns into a philosophical question. For some, the rationale of human space exploration is primarily related to high-value, near-term technological spinoffs, or the economic promises of soon-to-be accessible natural resources. The growing share of private companies involved in spaceflight often justifies their activities by the extensive possibilities of exploiting minerals and metals, and thus being able to address the ecological crisis on Earth. Others also invoke exploitation of space resources as a way of reducing the environmental cost of human activities on Earth, reconciling the words sustainable and economic development for future generations (39). As we have seen so far, reflection on deep space travel brings us to address ethical and philosophical questions such as human engineering (40), and the selection of phenotypes or genotypes of the terrestrial inhabitants. It further raises important questions about the future of sub-populations of astronauts derived from generations of humans after living in space. Therelationship between human populations that will not only differ in their phenotype (as evolution has to deal with contingency, and the evolution of different populations are likely to differ), but also in the way they view humanity's place in the cosmos. Astronauts have reported a shift in their relationship with Earth after a spaceflight. They specifically report that viewing the Earth from outer space increased their appreciation of its inestimable value and fragility (41). As developed over the past 30 years by Frank White in his Hypothesis of the Cosma, a cognitive shift in awareness toward Earth, named as the overview effect, will likely occur in the minds of deep space travelers.

Every evolutionary biologist has had to face criticism of his or her scientific questions. The lack of immediate deliverables applicable to short-term objectives is often cited in evaluations. This is due to a misunderstanding of the goals of evolutionary biology. Studying the short-term physiological adaptations to microgravity and the long-term consequences of living within a space environment using an evolutionary perspective is not incompatible, as both approaches are highly informative and relevant. However, we subscribe to the view that understanding the genomic, physiological and behavioral mechanisms underlying adaptations to new and contrasted environmental conditions must be placed in the light of evolution. Evolutionary biology is a field that attempts to understand a simple equation, i.e., how evolution actually finds a solution to an ecological problem. This is the question that life space science has tried to address: how do humans adapt to the space environment? By bringing current space research into the realm of evolutionary biology, we could generate new paradigms that will help humans to cope with deep space traveling. We are now entering a very exciting era during which a question such as this may be addressed.

## Author Contributions

FC wrote a first text, which was thereafter extensively drafted by AB, and further commented by CS.

## Conflict of Interest

The authors declare that the research was conducted in the absence of any commercial or financial relationships that could be construed as a potential conflict of interest.
